# Comparative Effects of Inulin with Different Polymerization Degrees on Growth Performance, Blood Trace Minerals, and Erythrocyte Indices in Growing-Finishing Pigs

**DOI:** 10.1007/s12011-016-0796-y

**Published:** 2016-07-19

**Authors:** W. Samolińska, E. R. Grela

**Affiliations:** Department of Bromatology and Food Physiology, Institute of Animal Nutrition and Bromatology, University of Life Science, Lublin, Poland

**Keywords:** Inulin Pigs, Iron, Copper, Zinc, Hematology indices

## Abstract

There are numerous reports of the effect of inulin on the bioavailability of mineral compounds. However, there are no conclusive reports concerning its beneficial impact (or lack thereof) in the case of such essential trace elements as iron, copper, or zinc. The aim of the study was to compare the effects of inulin addition with different degrees of polymerization (DPs) on growth performance in fatteners as well as on blood plasma concentrations of iron, copper, and zinc and selected hematological indices. The experiment was conducted throughout the fattening period (up to a body weight of approximately 115 kg) on 112 weaners with an initial weight of 25.0 ± 0.5 kg divided into 7 groups. The first group served as a control, while the other groups received increasing doses (1, 2, and 3 %) of standard inulin (SI; DP_av_ ≥ 10) or long-chain inulin (LCI, DP_av_ ≥ 23) in complete mixtures. Compared with the control, the supplementation of the mixtures with inulin increased the average daily gains, the final body weight, and the plasma content of trace elements (*P <* 0.05). An increased plasma zinc concentration was noted after application of inulin with a lower polymerization degree (*P <* 0.05). In turn, at a higher inulin polymerization degree, a higher final body weight and increased copper (*P <* 0.05), iron (*P* < 0.1), hemoglobin, mean corpuscular hemoglobin concentration (MCHC), and packed cell volume (PCV) levels were detected in animal blood (*P <* 0.05). The inulin addition was found to have modified the analyzed indices, and the optimal supplementation level was estimated at 20  g·kg^−1^ diet. Inulin with the higher DP exerted a more pronounced effect on the analyzed properties.

## Introduction

Inulin is a mixture of oligomers and linear fructose polymers with a varying degree of polymerization (DP) ranging from 2 to ca. 65 units with an average degree of polymerization (DP_av_) = 12 [1, 2]. In the gastrointestinal tract, inulin is a hydrolysis and fermentation substrate for the beneficial intestinal microbiota and increases the abundance of bacteria, primarily from the genus *Bifidobacterium* [3] and some *Lactobacillus* species [4, 5]. The molecule length is important for the technological and prebiotic properties of inulin [6–8] and depends on the source and extraction processes [9]. For instance, the number of fructose units and the polymerization degree (DP, 2–10; average 5) are reduced in the oligofructose obtained after partial enzymatic hydrolysis of chicory inulin [10]. The differences in the polymerization degree in fructans may affect not only their physicochemical and technological properties but also their biological traits. As shown by the investigations conducted by van de Wiele et al. [6], application of inulin was accompanied by increased production of short-chain fatty acids (SCFAs), greater *Bifidobacterium* abundance, and a stronger bifidogenic effect than that noted upon application of oligofructose, which is characterized by a lower polymerization degree. Inulin has been shown to be suitable for enhancement of the bioavailability of mineral compounds [11–15]. This is associated with stimulation of SCFA production by microorganisms, which reduce the pH of the intestinal tract, thereby increasing the solubility of minerals. Similarly, enhanced proliferation of intestinal epithelial cells in response to SCFA formation leads to an increase in the area available for mineral absorption. The bioavailability of mineral compounds can also be changed by the expression of nutrient transport proteins or regulatory genes involved in the absorption process [12, 16–19].

Among trace metals, iron, copper, and zinc are regarded as essential for the organism function. They are present in normal tissues at a relatively constant level, and their deficiency causes similar structural and physiological disorders both in various animal species and in humans [20]. Maintenance of homeostasis of these elements in the organism strongly influences erythropoietic processes and, consequently, red blood cell indices [21]. Strong antagonistic or synergistic interactions have been observed between these metals, including competitive inhibition of their bioavailability and transport in the gastrointestinal tract, which can simultaneously be reflected in their blood concentrations [22–26]. Blood serum and plasma are frequently tested for assessment of the normal trace element supply of the organism. The element content in the organism is a result of a dynamic balance between the demand and the amount supplied and the capability of elimination thereof [27, 28].

Until now, investigations of the inulin application in the nutrition for various animal species have yielded many ambiguous results concerning its impact (or lack thereof) on the bioavailability of essential trace elements, e.g., iron, copper, and zinc [13–15, 18, 19, 29–36]. This may be related to the application of inulin with different degrees of polymerization, which was emphasized in investigations conducted on piglets by Yasuda et al. [37] and Patterson et al. [38]. The available literature provides few reports of the inulin supplementation effects in older pigs’ diet [39]. This aspect may be interesting, as the effect of inulin in older animals may differ from that noted in piglets.

Hence, the aim of the study was to evaluate the effects of the inulin polymerization degree and its increasing doses supplemented in mixtures for pigs throughout the fattening period on growth performance as well as plasma iron, copper, and zinc levels and the related erythrocyte indicators.

## Material and Methods

### Animals, Diets, and Experimental Design

The experiment was conducted on 112 crossbreed weaners (Polish Landrace × Polish Large White) × Duroc with an initial weight of 25.0 ± 0.5 kg, divided into 7 equinumerous groups (16 pigs per group). Four animals per pen (two gilts + two barrows) were kept. The animals were fattened for 105 days. Each pen was equipped with a stainless steel self-feeder and a nipple drinker. Pigs had free access to feed and drinking water throughout the experimental period. Fatteners were fed ad libitum with complete diets, i.e., grower (25–70 kg) and finisher (71–115 kg). The hygienic conditions, i.e., the temperature, relative humidity, and cooling were the same for all the groups.

The supplementation with increasing doses of 10, 20, and 30 g per 1 kg of the feed mixture of two types of inulin extracted from chicory (*Cichorium intybus* L.) roots, differing in the DP, i.e., standard inulin (SI group, Orafti® GR, DP_av_ of inulin ≥ 10) or long-chain inulin (LCI group, Orafti® HPX, DP_av_ of inulin ≥ 23) was an experimental factor. The control group in the investigations was not supplemented with inulin. Commercial inulin replaced cornstarch in the control diet. The dietary composition and analysis are presented in Table 1.

**Table 1:** Ingredients and chemical composition of the experimental diets (as fed basis)

Diet type	Control	Standard inulin (SI)/long-chain inulin (LCI)			Control	Standard inulin (SI)/long-chain inulin (LCI)		
Inulin supplementation	0 g	10 g	20 g	30 g	0 g	10 g	20 g	30 g
Feeding phase	Grower (25–70 kg)				Finisher (71–115 kg)			
Ingredients (%)								
Wheat	30.0	30.0	30.0	30.0	25.0	25.0	25.0	25.0
Barley	37.5	37.5	37.5	37.5	53.1	53.1	53.1	53.1
Soybean meal (460 g/kg CP)	24.2	24.2	24.2	24.2	15.0	15.0	15.0	15.0
Soybean oil	1.2	1.2	1.2	1.2	0.5	0.5	0.5	0.5
Corn starch	3.0	2.0	1.0	0,0	3.0	2.0	1.0	0.0
Premix^a^	2.0	2.0	2.0	2.0	1.5	1.5	1.5	1.5
Inulin	0.0	1.0	2.0	3.0	0.0	1.0	2.0	3.0
Monocalcium phosphate	0.4	0.4	0.4	0.4	0.3	0.3	0.3	0.3
Limestone ground	1.2	1.2	1.2	1.2	1.1	1.1	1.1	1.1
Sodium chloride	0.3	0.3	0.3	0.3	0.3	0.3	0.3	0.3
L-Lysine	0.2	0.2	0.2	0.2	0.2	0.2	0.2	0.2
Total	100.00	100.00	100.00	100.00	100.00	100.00	100.00	100.00
Chemical composition (g·kg^−1^)								
ME MJ/kg^b^	13.14	13.14	13.13	13.13	12.91	12.90	12.88	12.87
Crude protein (g)	171.3	171.2	171.1	171.1	158.3	158.1	158.2	158.1
Lysine (g)	10.8	10.7	10.7	10.6	8.83	8.83	8.83	8.82
Methionine + cysteine (g)	5.82	5.81	5.79	5.79	5.43	5.43	5.42	5.42
Total calcium (g)	7.12	7.12	7.11	7.12	6.45	6.45	6.45	6.44
Total phosphorus (g)	5.07	5.07	5.06	5.06	4.87	4.87	4.87	4.87
Total iron (mg)	106.56	105.44	106.5	105.37	98.81	98.64	98.57	98.72
Total zinc (mg)	107.38	106.41	106.83	106.9	99.01	98.99	99.19	98.81
Total copper (mg)	7.87	7.79	7.71	7.9	6.8	6.72	6.73	6.79

*SI* standard inulin (Orafti® GR contained ∼92 % inulin, ∼8 % glucose, fructose, and sucrose (solubility < 12 g L^−1^ and DP ≥ 10) (Beneo-Orafti, Tienen, Belgium), *LCI* long-chain inulin Orafti® HPX contained 100 % inulin (solubility < 5 g L^−1^ and degree of polymerization (*DP*) ≥ 23) (Beneo-Orafti, Tienen, Belgium)

^a^One kilogram of the premix contained vitamin A 500,000 IU; D_3_ 90,000 IU; E 5000 mg; K_3_ 90 mg; B_1_ 90 mg; B_2_ 300 mg; B_6_ 150 mg; B_12_ 1.5 mg; nicotinic acid 1500 mg; pantothenic acid 700 mg; folic acid 160 mg; biotine 5 mg; choline chloride 12 g; Mg 12.5 g; Fe 1.5 g; Zn 4 g; Mn 3.5 g; Cu 100 mg; I 75 mg, Se 20 mg, Co 25 mg

^b^Calculated according to the NRC [59]

All pigs used in this study were examined and considered clinically healthy by a veterinarian. The study was approved by the Local Ethics Committee on Animal Experimentation of the University of Life Sciences in Lublin, Poland.

### Growth Performance

During the experiment, the animals were weighed at the start and before slaughter. Feed intake was controlled individually by weighing portions for automatic feed in the pens. The feed-to-gain ratio (F/G) was calculated.

### Sample Collection and Chemical Analyses

The total protein content was determined in the mixtures (AOAC 2000 [40]). The amino acid contents were determined using an automatic amino acid analyzer (AAA 400, Ingos, Czech Republic) after previous acid hydrolysis with 6 M HCl (method 994.12, AOAC 2000). Sulfur amino acids (cysteine and methionine) were determined in a separate analysis as oxidized derivatives (cystic acid and methionine sulfone) derived through performic acid oxidation and next released from proteins in the process of classic acid hydrolysis [41].

Samples of the mixtures were dried at 100 °C for 24 h and ashed for 10 h at 550 °C. The ashed samples were dissolved in a nitric acid-perchloric acid mixture (1:1) and diluted with deionized water for mineral analysis. The Fe, Zn, Cu, and Ca contents were measured using flame atomic absorption spectrophotometry (FAAS) (Unicam 939/959AA-6300, Shimadzu Corp., Tokyo, Japan), according to the Polish Standard (PN-EN ISO 6869:2002 [42]), and the total P content was determined colorimetrically (PN-76/R-64781:1976 [43]) with a Helios Alpha UV–Vis apparatus (Spectronic Unicam, Leeds, UK).

Blood samples were taken from the external jugular vein of six clinically healthy animals (three gilts and three barrows) fasted overnight for 8 h, twice from each group, when the body weights were 50 and 100 kg. Blood samples for hematological analyses were collected in 2-ml Vacutest tubes with a K_3_EDTA anticoagulant (Vacutest Kima s.r.l., Arzergrande (PD), Italy). For biochemical assays, blood was sampled in 6-ml Vacutest tubes containing lithium heparin (Vacutest Kima s.r.l., Arzergrande (PD), Italy).

Whole blood was analyzed within 3 hours after sampling. After placement of the samples on the hematological mixer (UMH-5, Wigo, Pruszkow, Poland), the red blood cell (RBC) count and the hemoglobin content (HGB) were determined using a hematological analyzer ABACUS Junior Vet (Automatic cell counter, Diatron, Vienna, Austria). The packed cell volume (PCV) was determined with the microhematocrit method. The analyses performed also included determination of other parameters of the red blood cell system. The mean cell volume (MCV) indicates the volume of an average red cell in a sample expressed in femtoliters (fl) and calculated using the following formula: MCV (fl) = PCV × 1000 / RBC. Mean cell hemoglobin (MCH) represents the absolute hemoglobin amount in an average red cell in a sample in units of picograms (pg) per cell. The MCH is calculated from the hemoglobin and the RBC using the following formula: MCH (pg) = (HGB (g·dl^−1^) × 10) / RBC. The mean corpuscular hemoglobin concentration (MCHC) is the average hemoglobin concentration in red blood cells calculated as follows: MCHC (mmol·l^−1^) = HGB / PCV [44].

Plasma for analysis of the biochemical parameters was obtained by centrifugation of whole blood at 3000 rpm (603×*g*) for 15 min in a laboratory centrifuge (MPW-350R, MPW Medical Instruments, Warsaw, Poland) at a temperature of 4 °C. Plasma without hemolysis signs was analyzed within 4 h after sampling, and the iron, zinc, and copper contents were determined. The elements were determined in blood plasma with colorimetric methods according to the manufacturer’s protocol using reagent kits (BioMaxima, Lublin, Poland; Hydrex Diagnostics, Warsaw, Poland) and a random access biochemical analyzer Metrolab 2300 GL (Metrolab SA, Buenos Aires, Argentine). The intra-assay coefficients of variation (CVs) of the method declared by the manufacturer were < 4.5, 2.4, and <2.6 % for the iron, zinc, and cooper determinations, respectively. The analysis procedures were verified with the use of multiparametric control plasma (BioCal), as well as control plasma of a normal level (BioNorm) and a high level (BioPath) of elements (BioMaxima, Lublin, Poland; Hydrex Diagnostics, Warsaw, Poland).

### Statistical Analysis

All calculations were performed with statistical software package Statistica 10.0PL (StatSoft Inc. [45]). The normality and homogeneity of variances were tested using the Shapiro-Wilk and the Brown-Forsythe tests, respectively. For comparative purposes, two types of statistical analyses were performed using a general linear model (GLM).

1. The effects of supplemental dietary inulin on the Cu, Zn, and Fe plasma concentrations, red blood cell indices, and growth performance traits were analyzed using one-way analysis of variance (ANOVA; using Fisher test or Welch test):

$$ {\mathrm{Y}}_{ij}=\mu +{\mathrm{a}}_i+{\mathrm{e}}_{ij} $$

where Y_*ij*_ is the measured variable, *μ* is an overall mean, a_*i*_ is the dietary inulin effect (treatment), and e_*ij*_ is the random error.

Model 2. A two-way ANOVA was performed with the GLM procedure to test the main effects and their interaction. The following model was used:

$$ {\mathrm{Y}}_{ijk}=\mu +{\mathrm{A}}_i+{\mathrm{B}}_j+{\mathrm{A}\mathrm{B}}_{ij}+{\mathrm{e}}_{ijk} $$

where Y_*ijk*_ is the measured variable, *μ* is an overall mean, A_*i*_ is the effect of the *i*th type of the inulin extract, B*j* is the effect of the *j*th level of the inulin supplement, AB_*ij*_ is the interaction of A_*i*_ and B_*j*_, and e_*ijk*_ is the random error. Means were compared using Duncan’s multiple range test. All statements of significance are based on a probability <0.05 and *P* values between 0.05 and 0.1 were considered as a trend.

In order to check the correlations between the inulin addition to the mixtures and the magnitude of the growth performance, Cu, Zn, and Fe plasma concentrations, and red blood cell indices, we calculated Pearson correlation coefficients (*r*) for the increasing inulin levels (10, 20, and 30 g·kg^−1^ mixture) and Spearman rank correlation coefficients (*R*) for the use of inulin with the various degrees of polymerization (groups: SI, DP_av_ ≥ 10 or LCI, DP_av_ ≥ 23). The coefficient of determination (*r* ^2^) was calculated for evaluation of the correlations between the variables.

## Results

### Growth Performance

The inulin addition to the mixtures did not have an impact on the feed intake in the fatteners or on the feed-to-gain ratio throughout the feeding period (Table 2). In turn, an increase in the average daily weight gain was observed (one-way ANOVA, *P* = 0.041) as well as a strong tendency toward an increase in the total weight gain throughout the feeding period (one-way ANOVA, *P =* 0.061), compared with the control group. Inulin supplementation of the mixtures elevated the final body weight of the fatteners (one-way ANOVA, *P* = 0.035). The multivariate analysis of variance confirmed the significant effect of the degree of inulin polymerization (IDP) on the analyzed growth indices. The addition of the long-chain inulin (LCI; DP_av_ ≥ 23) exerted a more pronounced effect on the average daily weight gain (two-way ANOVA, *P* = 0.083) and increased the final body weight of the fatteners (two-way ANOVA, *P* = 0.048). The amount of the additive and the interaction between the two factors appeared to be statistically insignificant. The absence of such an interaction may indicate that the explanatory variables were uncorrelated in this experiment, and the differences in the level of one variable did not alter the other.

**Table 2:** Effect of inulin on growth performance of pigs during the fattening period

Variables	Experimental diet							Inulin degree of polymerization (IDP)		Dietary inulin level, g kg^−1^ (IL)			Pooled SEM	One-way ANOVA	Two-way ANOVA		
														Significance^a^			
	Control	+10 g inulin		+20 g inulin		+30 g inulin		SI	LCI	10	20	30		T	IDP	IL	IDP ×IL
		SI	LCI	SI	LCI	SI	LCI										
Initial body mass (kg)	25.2	25.4	25.3	25.2	25.1	25.4	25.3	25.3	25.2	25.4	25.2	25.4	0.096	–	–	–	–
Final body mass (kg)	112.3b	113.5ab	113.6ab	114.3ab	115.9a	114.7ab	115.5a	114.2b	115.0a	113.6	115.1	115.1	0.178	0.035	0.048	NS	NS
Feed intake, (kg)	2.75	2.73	2.74	2.81	2.74	2.83	2.81	2.79	2.76	2.74	2.78	2.82	0.016	NS	NS	NS	NS
Weight gain (kg)	87.1	88.1	88.3	89.1	90.8	89.3	90.2	88.8	89.8	88.2	90.0	89.8	0.192	0.061	NS	NS	NS
Average daily gain (g)	829b	839ab	841ab	849a	865a	850a	859a	846	855	840	857	855	1.821	0.041	0.083	NS	NS
F/G (kg·kg^−1^)	3.32	3.25	3.26	3.31	3.17	3.33	3.27	3.30	3.23	3.26	3.24	3.30	0.016	NS	NS	NS	NS

Data are the means of 16 pigs (eight barrows and eight gilts) per group. Lowercase letters mean statistical differences *P <* 0.05 and *P <* 0.1 was considered as a trend

*SI* standard inulin, *LCI* long-chain inulin, *SEM* standard error of the means, *T* Treatments, *F/G* feed-to-gain ratio, *NS* non-significant differences

^a^Significance of the effects of the inulin degree of polymerization (*IDP*), inulin level (*IL*), and interaction between the inulin degree of polymerization and inulin level (*IDP × IL*)

A low positive correlation was only found between the degree of inulin polymerization and the final body weight of the fatteners (*R* = 0.338; *P* = 0.041). The calculated coefficient of determination (*r* ^2^) indicated that, in 11.42 %, the linear variation of the growth performance indicator was determined by the degree of polymerization of inulin applied in the diet.

### Plasma Trace Minerals

In the present experiment, the concentrations of trace minerals determined in blood plasma and the erythrocyte indices were in the reference ranges for this animal species reported in available literature [44, 46, 47].

Compared with the control group, the supplementation of the mixtures with inulin modified the plasma iron concentration in the fatteners (Table 3). It increased by 30 % in the finishing pigs receiving 20 g of long-chain inulin (one-way ANOVA, *P =* 0.046). The multivariate analysis also confirmed the tendency toward an effect of LCI on the plasma iron level, which was greater than the impact exerted by inulin with a lower degree of polymerization (two-way ANOVA, *P =* 0.074). Higher plasma levels of this element, i.e., from 5 to 13 % higher than in the control, were also detected in the other groups, but the differences were not statistically significant. Inulin influenced the plasma copper content only in the first phase of the fattening period (Table 3) (one-way ANOVA, *P =* 0.044), and this was primarily observed in the case of the LCI supplementation (two-way ANOVA, *P =* 0.020). The greatest changes were noted in the plasma zinc concentration throughout the feeding period (one-way ANOVA, *P <* 0.05). In comparison with the control group, an elevated level of this element was detected in the inulin-supplemented groups, except for that receiving LCI (DP_av_ ≥ 23) at the doses of 10 and 20 g·kg^−1^ of the mixture. Simultaneously, slightly higher iron concentrations were noted in these groups. The analysis of the significance of the linear model variables (IDP; IL) confirmed the effect of the degree of inulin polymerization on the plasma zinc content in the first and second phase as well as the entire fattening period (two-way ANOVA, *P* < 0.05). Additionally, the inulin dose had a significant effect on the concentration of this element in the first phase of the fattening period as well as throughout the fattening period. Furthermore, a combined effect of these two variables on the blood plasma zinc content was observed in the first phase of the fattening period (two-way ANOVA, *P =* 0.042), and a tendency toward such changes was noted throughout the fattening period (two-way ANOVA, *P =* 0.077). The calculated Spearman rank correlation coefficient (*R*) indicated a moderate negative correlation between the inulin polymerization degree and plasma zinc levels (*R* = −0.439; *P =* 0.002). The coefficient of determination (*r* ^2^) showed that 19.27 % of the variation in the plasma levels of the element was determined by the degree of inulin polymerization.

**Table 3:** Effect of inulin on the plasma iron, copper, and zinc content and hematological indices in growing–finishing pigs

Parameters	Fattening period	Experimental diet							Inulin degree of polymerization (IDP)		Dietary inulin level (g/kg) (IL)			Pooled SEM	One-way ANOVA	Two-way ANOVA		
															Significance^a^			
															T	IDP	IL	IDP × IL
		Control	+10 g inulin		+20 g inulin		+30 g inulin		SI	LCI	10	20	30					
			SI	LCI	SI	LCI	SI	LCI										
Trace elements																		
Plasma iron (μmol·l^−1^)	Growing	33.35 ±0.99	32.85 ±3.90	32.71 ±3.91	33.34 ±4.86	33.29 ±7.20	33.41 ±4.66	32.59 ±0.66	33.18 ±4.01	32.9 ±4.31	32.78 ±3.61	33.32 ±5.69	32.94 ±2.76	0.752	NS	NS	NS	NS
	Finishing	24.21b ±2.27	25.38ab ±4.45	27.34ab ±4.05	25.73ab ±1.77	31.41a ±2.27	25.60ab ±2.68	25.78ab ±4.82	25.55 ±2.49	28.18 ±4.04	26.34 ±3.98	28.57 ±3.13	25.59 ±3.22	0.722	0.046	0.074	NS	NS
	Total	28.13 ±5.44	29.09 ±5.95	30.03 ±5.07	29.54 ±5.47	32.35 ±5.28	28.95 ±5.35	29.18 ±5.30	29.20 ±5.34	30.52 ±5.17	29.56 ±5.44	30.94 ±5.39	29.07 ±5.12	0.714	NS	NS	NS	NS
Plasma cooper (μmol·l^−1^)	Growing	34.16bc ±1.83	34.43bc ±1.61	34.36bc ±1.55	35.24abc ±2.62	38.43a ±1.32	33.42c ±1.63	37.65a ±3.94	34.45b ±1.98	36.81a ±2.88	34.39 ±1.47	36.83 ±2.42	35.84 ±3.71	0.520	0.044	0.020	NS	NS
	Finishing	39.25 ±3.31	39.75 ±2.00	40.21 ±1.69	39.41 ±1.08	40.61 ±0.55	39.17 ±0.91	40.22 ±0.76	39.44 ±1.30	40.34 ±1.03	39.98 ±1.73	40.01 ±1.02	39.69 ±1.09	0.303	NS	NS	NS	NS
	Total	37.07 ±3.75	37.09 ±3.31	37.28 ±3.47	37.32 ±2.91	39.52 ±1.24	36.71 ±3.28	38.93 ±2.96	37.05 ±3.03	38.58 ±2.78	37.19 ±3.26	38.43 ±2.43	37.89 ±3.21	0.416	NS	0.082	NS	NS
Plasma zinc (μmol·l^−1^)	Growing	14.56B ±3.85	15.26A ±0.51	14.14 B ±0.35	15.94A ±1.56	14.22 B ±0.76	15.19A ±0.33	16.22A ±2.04	15.49a ±0.98	14.86b ±1.53	14.70b ±0.72	15.08ab ±1.46	15.78a ±1.55	0.323	0.005	0.032	0.029	0.042
	Finishing	14.62b ±3.14	16.02a ±1.29	14.06b ±0.27	15.30a ±0.90	14.90ab ±1.53	15.31a ±0.84	15.42a ±2.29	15.54a ±1.00	14.80b ±1.56	15.04 ±1.35	15.10 ±1.18	15.36 ±1.6	0.308	0.027	0.022	NS	NS
	Total	14.59B ±3.14	15.64A ±1.00	14.10B ±0.30	15.62A ±1.23	14.56B ±1.18	15.25A ±0.63	15.82A ±2.05	15.51a ±0.97	14.83b ±1.51	14.87b ±1.06	15.09ab ±1.28	15.56a ±1.54	0.221	0.008	0.025	0.041	0.077
Hematologic indices																		
RBC (10^12^·l^−1^)	Growing	7.08 ±0.31	6.92 ±0.14	7.18 ±0.15	6.89 ±0.17	6.87 ±0.05	7.23 ±0.37	6.88 ±0.16	7.02 ±0.29	6.97 ±0.19	7.05 ±0.19	6.88 ±0.13	7.05 ±0.33	0.049	NS	NS	NS	NS
	Finishing	7.13 ±0.39	7.22 ±0.76	7.44 ±1.22	7.04 ±0.85	7.06 ±0.13	7.01 ±0.74	7.20 ±0.99	7.09 ±0.71	7.24 ±0.84	7.33 ±0.95	7.05 ±0.56	7.11 ±0.81	0.136	NS	NS	NS	NS
	Total	7.10 ±0.32	7.09 ±0.57	7.33 ±0.88	6.97 ±0.57	6.98 ±0.14	7.12 ±0.55	7.04 ±0.68	7.06 ±0.54	7.11 ±0.63	7.21 ±0.72	6.97 ±0.42	7.08 ±0.60	0.549	NS	NS	NS	NS
HGB, (mmol·l^−1^)	Growing	7.74 ±0.14	7.97 ±0.51	7.74 ±0.09	7.85 ±0.48	7.72 ±0.56	7.74 ±0.99	8.13 ±1.43	7.84 ±0.65	7.89 ±0.90	7.85 ±0.35	7.79 ±0.48	7.94 ±1.16	0.139	NS	NS	NS	NS
	Finishing	7.32B ±0.35	8.12A ±0.95	8.89A ±0.83	8.16A ±0.59	8.36A ±0.29	7.48B ±0.68	8.09A ±0.76	7.92b ±0.75	8.44a ±0.89	8.50a ±0.92	8.26ab ±0.61	7.78b ±0.74	0.151	0.008	0.022	0.042	0.019
	Total	7.53b ±0.33	8.05ab ±0.74	8.40a ±0.85	8.01ab ±0.52	8.04ab ±0.43	7.61ab ±0.8	8.11ab ±1.06	7.89b ±0.69	8.18a ±0.88	8.22 ±0.79	8.03 ±0.53	7.86 ±0.94	0.102	0.044	0.047	0.098	NS
MCV (fL)	Growing	48.30 ±1.63	50.18 ±3.11	48.06 ±3.36	51.34 ±1.98	50.89 ±3.76	49.46 ±3.42	48.66 ±3.85	50.3 ±2.71	49.2 ±3.48	49.12 ±3.12	51.15 ±2.60	49.06 ±3.40	0.584	NS	NS	NS	NS
	Finishing	46.76 ±1.3	48.93 ±1.36	56.25 ±7.82	51.73 ±5.72	47.69 ±1.57	47.55 ±1.46	51.00 ±1.96	49.40 ±3.65	51.64 ±3.21	52.29 ±7.9	49.71 ±4.44	49.27 ±2.44	0.633	NS	NS	NS	NS
	Total	47.53 ±1.60	49.47 ±2.14	52.74 ±10.88	51.53 ±3.97	49.06 ±2.98	48.50 ±2.64	49.83 ±3.09	49.85 ±3.20	50.51 ±6.49	51.10 ±1.60	50.38 ±3.65	49.17 ±2.75	0.654	NS	NS	NS	NS
MCH (pg)	Growing	17.67 ±1.05	18.56 ±1.23	17.38 ±0.52	18.35 ±0.92	18.10 ±1.44	17.20 ±1.34	19.11 ±3.87	18.0 ±1.22	18.3 ±2.47	17.97 ±1.06	18.24 ±1.06	18.16 ±.87	0.352	NS	NS	NS	NS
	Finishing	16.56b ±0.68	18.10a ±0.40	19.85a ±5.11	18.81a ±1.96	16.78b ±0.59	17.20ab ±0.34	18.17a ±0.80	18.04 ±1.26	18.27 ±3.02	18.97 ±3.48	17.79 ±1.72	17.69 ±0.77	0.411	0.036	NS	NS	NS
	Total	17.11 ±1.01	18.30 ±0.80	18.79 ±3.86	18.58 ±1.44	17.34 ±1.16	17.20 ±0.90	18.64 ±2.63	18.01 ±1.01	18.28 ±2.72	18.54 ±2.69	18.00 ±1.42	17.92 ±2.04	0.271	NS	NS	NS	NS
MCHC (g·L^−1^)	Growing	365.53 ±17.27	369.58 ±2.66	362.24 ±21.55	357.13 ±8.58	355.33 ±7.90	343.14 ±9.82	389.96 ±47.19	357.03b ±11.65	371.26a ±33.57	365.91 ±14.31	356.36 ±7.65	368.74 ±38.86	4.737	0.059	0.042	NS	NS
	Finishing	354.05 ±13.29	370.02 ±16.92	356.63 ±71.02	369.21 ±3.93	351.72 ±9.19	361.69 ±8.26	356.07 ±4.91	366.97 ±40.03	354.80 ±37.55	363.05 ±48.33	360.46 ±49.66	358.88 ±6.97	6.777	NS	NS	NS	NS
	Total	359.79 ±15.53	369.83 ±12.06	359.03 ±51.82	363.17 ±49.15	353.27 ±8.17	354.61 ±11.30	373.01 ±35.96	362.22 ±29.81	362.28 ±35.94	364.43 ±36.58	358.55 ±35.53	363.81 ±27.45	4.191	NS	NS	NS	NS
PCV (l·l^−1^)	Growing	0.34 ±0.01	0.35 ±0.02	0.35 ±0.02	0.35 ±0.02	0.35 ±0.02	0.36 ±0.04	0.33 ±0.01	0.35 ±0.03	0.34 ±0.02	0.35 ±0.02	0.35 ±0.02	0.35 ±0.03	0.004	NS	NS	NS	NS
	Finishing	0.33b ±0.02	0.35ab± ±0.04	0.39a ±0.07	0.37a ±0.09	0.34ab± ±0.02	0.33b ±0.03	0.37a ±0.04	0.35b ±0.04	0.37a ±0.05	0.37 ±0.06	0.36 ±0.05	0.35 ±0.03	0.009	0.037	0.041	0.089	NS
	Total	0.34 ±0.01	0.35 ±0.02	0.37 ±0.03	0.36 ±0.05	0.34 ±0.02	0.35 ±0.03	0.35 ±0.01	0.35 ±0.03	0.35 ±0.02	0.36 ±0.02	0.35 ±0.03	0.35 ±0.02	0.006	NS	NS	NS	NS

Data are the means ± standard deviation of six pigs (three barrows and three gilts) per group. Lowercase letters mean statistical differences *P <* 0.05. Uppercase letters mean statistical differences *P <* 0.01. *P <* 0.1 was considered as a trend

*RBC* red blood cell, *HGB* hemoglobin, *MCV* mean corpuscular volume, *MCH* mean corpuscular hemoglobin, *MCHC* mean corpuscular hemoglobin concentration, *PCV* packed cell volume, *SI* standard inulin, *LCI* long-chain inulin, *SEM* standard error of the means, *T* treatments, *NS* non-significant differences

^a^Significance of effects of inulin degree of polymerization (*IDP*), inulin level (*IL*), and interaction between inulin degree of polymerization and inulin level (*IDP × IL*)

### Erythrocyte Indices

The inulin addition to the mixtures did not induce changes in the levels of such erythrocyte indices as the red blood cell (RBC) count and the mean corpuscular volume (MCV) (Table 3). In contrast, the supplementation with this additive altered the hemoglobin content. Compared with the control, the greatest changes were exhibited by the animals in the second phase of the fattening period, when the hemoglobin level increased by 11–21 % (one-way ANOVA, *P =* 0.008), with the exception of the group supplemented with 30 g of standard inulin (SI), in which no hemoglobin increase was noted. The multivariate analysis of variance confirmed the impact of both the inulin polymerization degree and its dose as well as the combined effect of these two factors on the analyzed variable (two-way ANOVA, *P <* 0.05). The long-chain inulin increased the hemoglobin level more efficiently (by 11 %) in comparison with the standard inulin. A reverse correlation between the inulin supplementation and the blood hemoglobin level was also noted. The addition of 10 g·kg^−1^ inulin to the mixture increased the hemoglobin content by 11 %, relative to the highest inulin dose (30 g·kg^−1^). Throughout the fattening period, a similar, although lesser, effect of the inulin addition on the hemoglobin content was noted (one-way ANOVA, *P =* 0.044). The analysis of variance taking into account the polymerization effect confirmed the stronger impact of the long-chain inulin on this blood index (two-way ANOVA, *P =* 0.047). In turn, the effect of the inulin dose was weaker and only a tendency toward increased hemoglobin synthesis at the lower inulin dose added to the mixture was found (two-way ANOVA, *P <* 0.1). Similarly, the calculated Pearson correlation coefficient (*r*) between the dose of the inulin addition and the hemoglobin concentration confirmed their low negative correlation (*r* = *-*0.369; *P =* 0.010). The coefficient of determination (*r* ^2^) showed that 13.61 % of the hemoglobin level variation was modified by the added inulin dose.

The mean cell hemoglobin (MCH) index was modified by the inulin supplementation of the mixtures only in the second phase of the fattening period, compared with the control (one-way ANOVA, *P =* 0.036). In the first phase of the fattening period, a positive effect of the degree of inulin chain polymerization on the mean corpuscular hemoglobin concentration (MCHC) was observed (two-way ANOVA, *P* = 0.042).

Compared with the control, the packed cell volume (PCV) was significantly higher (one-way ANOVA, *P =* 0.037) in the blood of animals supplemented with 20 g of SI or 10 and 30 g of LCI only in the second phase of the fattening period. In this phase of fattening, there was a positive effect of the degree of inulin chain polymerization on PCV (two-way ANOVA, *P =* 0.041). Simultaneously, a tendency toward a decrease in this hematological index at the increasing inulin doses was observed (two-way ANOVA, *P <* 0.1).

## Discussion

Supplementation with inulin as a fattener feed additive increased the daily gains and the final body weight (*P <* 0.05). The literature presents many investigations of inulin supplementation in the diet for many animal species. However, the results obtained are often ambiguous in terms of the effects on the growth performance. This may be associated with the diverse types of inulin differing in the polymerization degree or mixtures thereof with oligofructose (Table 4). A beneficial effect of inulin supplementation on the final body weight was reported in investigations conducted on pigs [30], broiler chicken [33], and fish [36]. In the present research, a stronger impact on the final body weight in the fatteners was noted after addition of the long-chain inulin (group LCI; DP_av_ ≥ 23), while there were no concurrent changes in the feed intake in the experimental groups. This may suggest improvement of utilization of nutrients from the mixtures supplemented with the long-chain inulin reflected in the final body weight. According to the literature, inulin-induced changes in bacterial populations can exert a beneficial effect on the digestion and absorption processes, including the activity of intestinal enzymes, nutrient metabolism, and intestinal histomorphology, thereby contributing to the growth performance [12, 16, 17].

**Table 4:** Effect of inulin on growth performance, concentration of trace elements, and hematology indices in animal models

Animal model/age or body weight at start	Dose inulin	Inulin form	Duration of administration	Results	Reference
Fish					
Nile tilapia juveniles/42–47 g	2.5 and 5 g kg^−1^ diet	Prebiofeed 88: chicory inulin, 90 % inulin, 10–40 DP	8 weeks	Final weight, feed intake, ↑ FCR, ↓ HGB, → PCV, → RBC count, ↑ serum iron content, ↑	Tiengtam et al. [36]
Great sturgeon juveniles/16.14 ± 0.38 g	10, 20, and 30 g kg^−1^ diet	Raftifeed IPS: chicory inulin, 90–95 % inulin, 3–65 DP, (DP_av_ = 10)	8 weeks	HGB, PCV, MCV (1.0, 2.0% inulin in diet), → HGB, PCV, MCV (3.0% inulin in diet), ↓ MCH, → MCHC, → RBC count, →	Ahmdifar et al. [32]
Poultry					
Male broiler chickens/day old	5 and 10 g kg^−1^ diet	Orafti GR: chicory inulin, ∼92 % inulin (DP_av_ ≥ 10)	42 days	BWG (0.5% inulin in diet), → BWG (1% inulin in diet), ↑ FCR (0.5% inulin in diet), → HGB, → RBC count, → FCR (1% inulin in diet), ↓	Nabizadeh et al. [33]
Male broiler chickens/1 day old, 40.74 ± 0.3 g	5, 10, and 15 g kg^−1^ diet	Commercial inulin from chicory: 92 % inulin, DP_av_ ≥ 10	42 days	BWG, → FCR, →	Huang et al. [35]
Rats					
Male rats/6 weeks old, ∼170 g	50, 100, and 200 g kg^−1^ diet	Commercial inulin from chicory	21 days	Feed intake, BWG (20% inulin in diet), ↓	Levrat et al. [11]
Male rats/ 2, 5, 10, and 20 months old	37.5 g kg^−1^ diet 75 g kg^−1^ diet	Raftaline: chicory inulin, 3–50 DP (DP_av_ = 9)	4 days 26 days	Food intake and BWG, → Plasma Cu, ↑ Plasma Zn, ↑ Cu and Zn intestinal absorption and retention, ↑	Coudray et al. [13]
Male rats/6 weeks old, 116 ± 2 g	100 g kg^−1^ diet	Raftilose synergy 1: chicory inulin, combination (1:1) of long-chain inulin (DP 10–65, DP_av_ = 25) and oligofructose (DP 2–8, DP_av_ = 4)	15 days	BWG, ↓ Food intake, ↓ Food efficiency, → Fe, Cu and Zn intestinal absorption, ↑	Lobo et al. [15]
Male rats/160 g	100 g kg^−1^ diet	Commercial inulin from chicory: 3–50 DP (DP_av_ = 9)	10 days	Apparent Fe and Cu absorption, ↑ Apparent Zn absorption, → Plasma Cu, Zn and Fe, →	Lopez et al. [29]
Pigs					
Pigs/51.1 ± 0.41 kg	0 and 50 g kg^−1^ diet	FibrulineR: chicory inulin, 90 % inulin	5 weeks	BWG,↑ serum Fe, → HGB, → PCV, → RBC count, →	Jayasooriya et al. [30]
Weanling pigs/9.23 ± 0.03 kg	20 and 40 g kg^−1^ diet (experiment diets without inorganic iron)	Raftilose synergy 1: chicory inulin, combination of long-chain inulin (DP 10–65, DP_av_ = 25) and oligofructose (DP 2–8, DP_av_ = 4)	5 weeks	BWG, → feed intake, → final body weight, → HGB, ↑ PCV, →	Yasuda et al. [14]
Anemic male weaner piglets/4-week old	40 g kg^−1^ diet	Raftilose synergy 1: chicory inulin, combination of long chain inulin (DP 10–65, DP_av_ = 25) and oligofructose (DP 2–8, DP_av_ = 4)	5 weeks	Iron absorption, → HGB, → HRE, → Serum Fe, → TIBC, → Transferrin saturation, →	Patterson et al. [31]
Male piglets/8.6 ± 1.7 kg, 28 days old	15 g kg^−1^ diet	Raftiline® HP: chicory inulin, 100 % long-chain inulin (DP_av_ ≥ 23.0)	11 days	Body weight, → Liver Cu and Fe concentration, ↑ Liver Zn concentration, →	Taranu et al. [34]

*BWG* body weight gain, *DP*_*av*_ average degree of polymerization, *FCR* feed conversion ratio, *HGB* hemoglobin, *MCV* mean corpuscular volume, *HRE* total body hemoglobin Fe, *MCH* mean corpuscular hemoglobin, *MCHC* mean corpuscular hemoglobin concentration, *PCV* packed cell volume, *RBC* red blood cell, *TIBC* total iron binding capacity, *↑* increase, *→* no significant differences, *↓* decrease

The results obtained in this study confirm the significant role of the inulin addition in increasing the zinc, copper, and iron concentration in fatteners’ blood plasma (*P <* 0.05). The reported changes were more pronounced in the case of zinc rather than copper and iron, which may have resulted from a better supply of these trace elements in the organism. In the case of iron, the regulation of absorption from the intestinal epithelium into the blood largely depends on the amount of iron stored in the organisms and the erythropoiesis rate. An increasing iron demand at low amounts stored leads to increased absorption of this element [44]. The stimulatory effect of inulin on Fe, Zn, and Cu absorption and on the plasma levels of these biometals was reported previously [13, 15, 29, 36]. The mechanism of improved availability of macroelements and microelements by fructans is associated with fermentation thereof by the intestinal saprophytic microbiota and formation of organic acids (primarily SCFA), which lower the pH of the intestinal contents and increase the solubility of mineral compounds [48]. Increased absorption may also result from improved integrity of the intestinal epithelium barrier providing protection against pathogenic bacteria, increased proliferation of epithelial cells, and reduced inflammation in response to SCFA production [12, 17, 48]. Simultaneously, inulin administration is accompanied by increased bacterial hydrolysis of phytates, which contain complexed zinc, iron, and copper [29, 48]. Phytates are present in substantial amounts in grains, cereal bran, and legumes, i.e., the basic feed components of feed mixtures for pigs. Yasuda et al. [19] and Tako et al. [18] reported a positive effect of inulin on the expression of genes encoding Fe transporters, enzymes, and ferritin in intestinal enterocytes and its inhibiting effect on inflammation-related genes, which exerts a positive influence on iron metabolism as well. Yasuda et al. [19] also found that the inulin-induced improvement in iron utilization was independent of the inulin chain polymerization degree.

In the study, there were differences between the effects of the standard inulin and the long-chain inulin on the plasma content of the analyzed trace elements. Other animal investigations focused on assessment of Fe, Zn, or Cu absorption revealed either beneficial effects or absence of an impact of these fructans on Fe, Zn, or Cu absorption [13, 15, 29, 31] (Table 4). The differences between the results obtained in this study and literature reports may be associated with interspecific differences, animal age, a shorter period of inulin supplementation of the feed, and the degree of inulin polymerization. Alles et al. [49] and van de Wiele et al. [6] indicate that the degree of polymerization (DP) largely determines the site of fructan fermentation in the gastrointestinal tract. Fructans with a low polymerization degree undergo relatively fast microbial fermentation, while long-chain fructans are more resistant to fermentation and undergo the process only in the end parts of the gastrointestinal tract [6, 38, 50]. Kleessen et al. [4] showed that the effect of fructans on the level of short-chain fatty acids produced in the cecum and colon depended on the chain length. In their investigations based on addition of a mixture (1:1) of long-chain inulin (DP_av_ = 25) and oligofructose, Yasuda et al. [51] determined mainly inulin decomposition in the jejunum, cecum, and colon in piglets. The process was characterized by the greatest dynamics in the cecum. Alles et al. [49] and Patterson et al. [38] reported that fructans with varied degrees of polymerization stimulated the growth and activity of various *Bifidobacterium* and *Lactobacillus* species present in the entire gastrointestinal tract. Short-chain fructans were fermented by a greater number of *Bifidobacterium* species [52]. This slightly different fermentation site in the gastrointestinal tract, dependent on the polymerization degree, and the influence of inulin on the intestinal microbiota can have varying effects on the absorption of trace elements and their blood concentration. In the present study, throughout the animal fattening period, inulin with the lower polymerization degree (SI; DP_av_ ≥ 10) increased the blood plasma zinc content (*P =* 0.025). In monogastric animals, mineral compounds are mainly absorbed in the small intestine and, to a lesser extent, in the colon [53]. Zinc is absorbed in the duodenum and jejunum, and phytic acid is the basic inhibitor of absorption of this element [54]. These complexes can be hydrolyzed by the intestinal microbiota stimulated by the presence of inulin, which contributes to the release of bound zinc. Given the highly limited zinc absorption in the large intestine, release thereof through fermentation has biological relevance probably only at application of standard short-chain inulin, which is more easily degraded by gastrointestinal bacteria in the upper intestine parts. The results presented in this study also indicate a plasma zinc concentration increasing together with the inulin dose, particularly in the first phase of the fattening period (*P =* 0.029), which implies an interaction between the inulin dose and the polymerization degree (*P =* 0.042). Similarly, Levrat et al. [11] confirmed the linear relationship between the inulin dietary content (0, 5, 10, and 20 %) and Ca and Mg absorption in the rat cecum. On the other hand, increased zinc absorption may result in reduced iron and copper absorption and, consequently, iron metabolism disturbances (reduced concentrations of hemoglobin and hematocrit), which was also noted in the present study.

After the addition of inulin with the longer fructose chain (LCI; DP_av_ ≥ 23), a reverse situation was observed, i.e., increased amounts of copper (*P <* 0.05) and iron (*P <* 0.1) in the plasma. This is probably related to the site of long-chain inulin fermentation and absorption of these elements. In contrast to zinc, both iron and copper can partially be absorbed in the large intestine [55–57]. In their observation of enhanced Cu and Fe absorption in rats receiving inulin supplementation, Lopez et al. [29] confirmed that it primarily reflected the increased absorption of these microelements in the large intestine.

Assessment of the hemoglobin level is an efficient indicator of the iron content in the organism while monitoring the effects of nutritional intervention. Our results of the evaluation of erythrocyte indices have revealed an increase caused by the inulin addition in both the hemoglobin content and the MCH and PCV levels, compared with the control (*P <* 0.05), which may be associated with the enhanced absorption of the elements involved in erythropoietic processes (copper, iron, zinc). A similar positive effect of inulin supplementation has also been reported by Yasuda et al. [14] (Table 4). The investigators found a 15 % increase in the hemoglobin concentration in piglets’ blood (*P <* 0.01) in inulin-supplemented groups, compared with the control group. A similar impact of inulin addition has been noted in experiments on calves [58]. The authors explained the positive influence of inulin on the red blood cell system by better absorption of iron resulting from optimization of conditions prevailing in the intestine and enhanced utilization of iron in hemoglobin synthesis. Our findings related to the assessment of the hemoglobin content and the PCV and MCHC indices have demonstrated a more beneficial effect of the long-chain inulin addition (*P <* 0.05). These indices are associated with the blood hemoglobin concentration, which is a most common criterion in diagnosis of iron deficiency. In investigations conducted on other animal species, no such effect of inulin on erythrocyte indices was reported [30–33] (Table 4). In the present study, in particular in the second phase of the fattening period, a negative impact of the increasing inulin doses (*r* = −0.369; *P =* 0.010) on the hemoglobin concentration (*P =* 0.042) and the PCV value (*P =* 0.089) was observed. Similar effects were noted by Ahmdifar et al. [32] for inulin supplementation in sturgeon feed. This may have been caused by enhanced zinc absorption at the higher doses of inulin. Zinc induces production of metallothionein in enterocytes, and the protein binds copper, thereby preventing absorption thereof, which exerts an effect on iron metabolism and the level of erythrocyte indices [53].

## Conclusions

The inulin supplementation effectively modified the evaluated indices, and 20 g·kg^−1^ diet was found to be an optimal dose. A clearly more beneficial effect was exerted by the long-chain inulin. A more pronounced effect of standard inulin on the final body weight, blood copper and iron concentrations, and erythrocyte indices was noted.

The different inulin effects depending on its polymerization degree suggest a necessity to carry out further investigations on the combined application thereof in animal nutrition. For evaluation of the trace minerals status, more sensitive indicators such as molecular biomarkers should additionally be used. This will contribute to detailed assessment of induction of a possible synergistic effect on trace element supply in the organism and relevant red blood cell indices.
